# Energy transition, rural transformation and local land-use planning: Insights from Ontario, Canada

**DOI:** 10.1177/25148486211024909

**Published:** 2021-08-09

**Authors:** Kirby Calvert, Emily Smit, Dan Wassmansdorf, John Smithers

**Affiliations:** University of Guelph, Canada

**Keywords:** Renewable energy, energy transition, land-use plans, discourse analysis, rural geography

## Abstract

A transition toward decentralized and land-intensive renewable energy production systems is one among many factors re-shaping rural areas, leading to reimaginations and contestations. Especially in the Global North, the rural narrative now includes not just rural ‘production’ but also the ‘consumption’ of rural amenity and experience. Previous research into public attitudes toward renewable energy correlates the former with positive attitudes to renewable energy, and the latter with negative attitudes toward renewable energy. Territorial structures, such as official land-use plans, reflect dominant discourses and narratives that shape ongoing rural transformation. The purpose of this work is to understand the extent to which, if at all, those correlations at the individual level between landscape conceptualizations and sentiment toward renewable energy are manifest in territorial structures. In what ways are energy transitions present in rural land-use plans and planning systems? Is there a relationship between how rural landscapes are conceptualized and how energy transitions are framed and addressed, in land-use planning systems? These questions are answered through a structured content and discourse analysis of 10 land-use plans of rural municipalities in southern Ontario; an agriculturally intensive region that hosts much of Ontario’s large-scale renewable energy systems. Correlations observed between landscape conceptualizations and sentiment toward renewable energy observed are not strongly reflected in land-use plans. Land-use plans in this region are not positioned to manage the place-based opportunities and impacts associated with renewable energy development. The research reveals an opportunity for rural land-use planning systems to more explicitly incorporate energy transitions in their evolving discourses, identities and development trajectories.

## Introduction

Prospecting for renewable energy (RE) resources, as with other natural resources, is predominantly a rural affair. The technologies at the forefront of the transition toward RE resources are decentralized, area-dependent and land-intensive. As such, RE resources are most easily accessible, technically speaking, in rural areas. The dependence of the RE sector on rural land and infrastructure may change with improvements to technologies that can harvest RE from existing infrastructure (e.g., building integrated systems), off-shore and even in outer space. But for the foreseeable future at least, the energy transition is yet another moving part in a broader and ongoing process of rural transformation. In many regions, rural spaces and places are being contested and (re)imagined – materially and discursively – irrespective of the RE transition, through ongoing processes of economic, social, political, environmental and technological change. Such processes of rural change, together with longstanding place-based narratives and local histories, provide both a conceptual and pragmatic context within which the prospect and process of energy transition must be viewed (Beckley, 2017; Huber and McCarthy, 2017; McCarthy, 2015; Naumann and Rudolph, 2020).

In light of the proliferation of RE systems across rural areas of the developed economies, a form of rural protectionism has emerged with calls to ‘safeguard’ countryside landscapes against RE development (Jefferson, 2018). From this perspective, ‘the visual intrusion of wind energy developments on rural landscapes, and the beginnings of visual intrusion of solar ‘arms’ and further transmission linkages as well, raise acute concerns about where the world is headed’ (Jefferson, 2018: 196) and a ‘sustainable future requires us to preserve scenic values and protect many rural landscapes’ (Jefferson, 2018: 191). Protectionist perspectives are understandable. Certainly, there is a need to understand energy transition as one specific driver of rural transformation and to address the impacts of energy transition on the rural places that many people call home. Calls for a ‘just’ energy transition, that explicitly address distributive, procedural and recognitional equity, demand such attention (see Williams and Doyon, 2019).

At the same time, there is a need for critical approaches that ask questions about the ‘kind’ of rural landscapes that are protected and for whom/what purpose (see Cowell, 2010; Naumann and Rudolph, 2020). We need to take seriously the fact that energy transition is entangled in the power dynamics of place-making (Bridge et al., 2013; Calvert, 2016; Murphy, 2015; Wolsink, 2020). Calls to protect ‘the rural’ are calls to protect a *particular idea* of ‘rural’. In other words, these calls are essentialist and essentializing discourses that conflate ideas about what *ought to be* considered appropriate or acceptable in a rural milieu with ideas about *what is and will always be* appropriate or acceptable in a rural context (Batel et al., 2015; see also Sherren, in press). Such discourses provide a frame through which RE futures in particular areas are negotiated and advanced (Wolsink, 2020). Here, we call attention to the fact that particular ideas about ‘rurality’, and established connections/livelihoods derived from the prevailing form and function of rural places, are entwined with social responses to RE infrastructure (Beckley, 2017; Naumann and Rudolph, 2020).

The purpose of this paper is to contribute to a small but growing body of scholarship that seeks to understand how rural imaginations and discourses are entangled with, and shaping, processes of energy transition in rural areas (see Phadke, 2011; van Veelen and Haggett, 2017; Rudolph and Kirkegaard, 2019; Batel, 2020). Our empirical focus is local land-use planning documents in an agriculturally intensive region of the Canadian province of Ontario, a region that has the most potential to host RE projects and, hence, contribute to energy transitions. Rural land-use planning documents offer insight into the dominant spatial representations and identities that are acting as levers of development control in general (Cadieux et al., 2013) and over RE more specifically. Indeed, land-use plans institutionalize and codify (dominant) representations of place and place-based identities through practices which implement and negotiate values and priorities; some of which are pursued, others not (Crawford and French, 2008; see also Anderson, 2010; Blok and Meilvang, 2015). Our empirical objective in this paper is two-fold: first, assess the extent to which rural land-use plans are identifying and managing (or not) the opportunities and challenges related to RE development; second, identify dominant discourses about rurality codified in those documents and situate those discourses in relation to energy transition to understand how rural land-use plans and discourses are (co)evolving with energy transition, or not. We suggest that these insights are critical to our understanding of the contested and evolving geographies of energy transition more generally.

The paper proceeds as follows. In the next section, we review key literature and establish the conceptual framework for the research; this is followed by a section that introduces the case study and describes the methods. A further section summarizes our key results, which are discussed in the penultimate section. We then conclude by illustrating the significance of rural land-use plans in the shaping of rural spaces and framing of energy transitions. 

### Constructing rural transformation and energy transition in the Global North

Our research resides at the intersection of scholarship in energy geographies and rural geographies (see also Naumann and Rudolph, 2020). For present purposes, we note three pertinent themes from rural geography that inform our appreciation of the place-based dynamics of the RE ‘project’ writ large: the social representation of ‘rural’ and ‘rurality’; the construction (and consumption) of amenity landscapes; and the emergence of the multi-functional countryside. We bring these themes into dialogue with literature on the spatial politics of energy transition. Combined, these perspectives help to interpret the politics of energy transition more effectively in rural areas of the Global North and provide a conceptual framework through which to examine energy transition in rural land-use plans.

### Rural in the mind’s eye

While the objective concept of rural as a land-use designation is amenable to simple description and measurement based on a selection of objective measures (population density, services, etc.), the ‘notion’ or experience of rural and rurality is a very different matter – one that lies at the heart of much of the contestation around rural spaces and places in the face of change. Early influential studies by Cloke (1997), Cloke and Milbourne (1992), and Halfacree (1993) have called attention to the role of social representation and social construction in influencing the dynamics of social, economic, political (and related environmental) processes underlying rural change and has proved especially helpful in unpacking the motives and actions of individuals and interested actor groups in the face of various specific change circumstances. In contrast to the descriptive crispness of statistical measures, Halfacree (1995) suggests that what it means for something or someone to ‘be’ rural is captured in the words and imagery used by people in everyday life – a matter of some importance in understanding the character of rural space and the manner in which rural change unfolds (Halfacree, 2006).

Batel et al. (2015) encourage analysts to go beyond individualist and socio-cognitive analytical approaches to people-place interactions and to recognize that ‘…people-place relations are shaped by different and competing representations, claims, and power relations … place attachments and identities are … *a socially constructed* “*way of seeing*”’ (Batel et al., 2015: 150). In other words, rural attachments and identities that factor into personal attitudes toward RE are intersubjective – e.g., they inform, and are informed by, collectivized narratives advanced through media representations, corporate/government/special interest branding materials and the like (Batel, 2020; Phillips et al., 2001). Furthermore, rural attachments and identities are power-laden to the extent that they are imbued with underlying values, invoked and mobilized in policy discussions and become codified into territorial structures that aim to order socio-spatial processes in particular places (Cadieux et al., 2013).

As noted earlier, rural spaces are being contested and (re)imagined – materially and discursively – irrespective of the RE transition, through ongoing processes of economic, social, political, environmental and technological change. The emergence and growth of two broad socio-economic and landscape transformations are especially notable for their implications on energy transition in the Global North: the emergence of the so-called ‘consumption countryside’ (Dwight-Hines, 2012; Kordel, 2016; Marsden et al., 1993) and the advancement of the notion of ‘multi-functionality’ as a means of achieving social and environmental sustainability in rural areas (Renting et al., 2008; Wilson, 2007).

### A consumption countryside

In its simplest form, the emergence of a consumption countryside implies a shift in economic and related social dominance away from traditional land-based production activities, namely, farming in Southern Ontario, to more consumption based, largely experiential, activities drawing on the appeal and marketability of either the natural environment or heritage assets or both (Hiner, 2014; Roberts and Hall, 2004; Woods, 2005). The emergence and growth of amenity-based modes of rural land and regional development is now well established as a driving force in the transformation of many rural regions, perhaps most clearly and compellingly recognized as an essential feature of the emergence of Wine Regions (Overton and Murray, 2011; Visentin and Vallerani, 2018). In these instances, it is the built features, community and/or landscape itself that is on offer, with the ability to command a higher value than its previous use could provide based on the attraction it holds for those who wish to ‘consume’ it. The scholarly literature has taken interest in two main forms of this phenomenon: rural tourism and (rural) amenity migration.

In the tourism realm, there is now a large body of scholarship that grows from both the farm diversification literature (Barbieri and Mahoney, 2009; Northcote and Alonso, 2010; Sharpley and Vass, 2006) and from the wider tourism literature (LaPan and Barbieri, 2014; Veeck et al., 2006). In the former, farm diversification has been long promoted as one available pathway of enterprise development for those farms wishing to move away from intensive production and the global food system and has been actively promoted and supported by state actors. In the latter, tourism writ large has long been promoted as a vitally important component of rural economic development – especially following the downturn in markets for natural resource commodities and the uncertainty of international markets for exports (Lupi et al., 2017). Tourism has emerged as a ‘go to’ strategy for adaptation in struggling rural communities and often embraced fully by those with natural environmental assets.

Although less ubiquitous, amenity-based migration is a cousin phenomenon to rural tourism but driven by somewhat different forces, featuring different discourses and producing different outcomes. As with tourism, the literature here is abundant concerning the factors driving this form of counter-urbanization, but two points offered by Argent et al. (2007) are particularly notable. First, development controls notwithstanding, amenity-rich rural areas continue to hold strong intrinsic appeal for large numbers of people who, if afforded the opportunity, would welcome the prospect of country living (Argent et al., 2014; Curry et al., 2001). High urban housing prices and ageing populations have resulted in notable retirement-related urban to rural migration patterns in many regions. Second, in-migrants to rural communities have been shown to have comparably greater financial power and a penchant for greater activism and/or a willingness to engage political processes in pursuit of a desired outcome (Abrams et al., 2012; Phadke, 2011; Tonts and Greive, 2002). Taken together such circumstances have become implicated in the so-called ‘gentrification of the countryside’ (Guimond and Simard, 2010; Phillips and Smith, 2018) and a discourse that, in the local food sector, has been characterized as ‘defensive localism’ (Winter, 2003; such an analogy seems applicable here also). As has already been experienced in agriculture, particularly animal agriculture, in some regions, the prospect of unwelcomed production-based intrusions into an amenity landscape and a ‘purchased lifestyle’ set up the possibility of contestation in the energy sector.

### A multi-functional rural

In large part, the concept of multi-functionality is intuitive in that it suggests a rejection of simple ‘single use categories’ in thinking about rural land and the many and varied demands individuals and society place on rural land. Coining of the term ‘multi-functionality’ in reference to rural land use has origins in the European agricultural policy realm but has since found wide application, at least conceptually, in sustainable rural systems scholarship since that time – particularly with reference to the trajectories and possibilities of farm-level change (Wilson, 2007) and the role of farming at a wider landscape level in relation to a broader notion of ‘services’ derived from agricultural land use (Wilson, 2008). The term seeks to capture contributions from farming that extend beyond the mere provision of food commodities to the industrial/conventional food system to include contributions to biodiversity retention, local food system security, farmland preservation, rural economic prosperity and rural community vitality (Dibden and Cocklin, 2009). More recently, these have expanded to include activities such as carbon sequestration and energy production. Although initially concerned with the nature and process of change at the farm level and with the ‘place’ of the farm within an evolving rural sector, the concept of multi-functionality is inherently spatial and scalar in nature (Wilson, 2008) and has been employed as a means of differentiating not just the range of contributions from farming at the farm level, but also the complexity of the countryside and region where a wider range of activities and actors come into view (Knickle and Renting, 2000) – a vantage point from which it is possible to see a matrix of rural development components and the ways that land-based production is (or is not) integrating.

**Figure 1.**
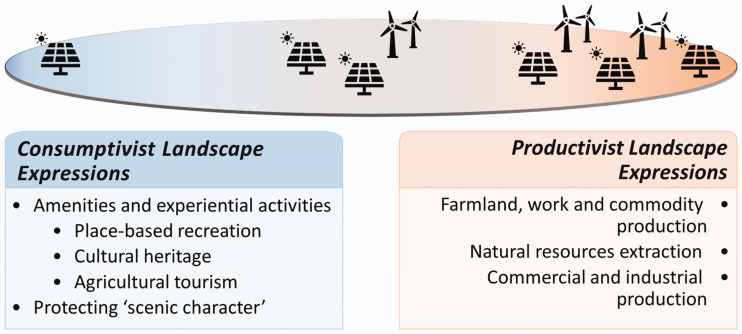
Prior research suggests that references to renewable energy transitions and infrastructure will be more prevalent and more prominent in rural plans that conceptualize and express their landscape predominantly in productivist terms. Conversely, references to renewable energy transitions and infrastructure will be less prevalent and less prominent in rural plans that conceptualize and express their landscape predominantly in consumptivist terms. Conceptually, the ‘multi-functional rural’ resides somewhere in the middle.

Holmes (2006) has applied, more explicitly, the concept of multi-functionality in a spatially extended context by suggesting the existence of a multi-functional rural transition built around three broad purposes of rural land and landscapes: production, consumption and protection. In differentiating the evident range of demands or trajectories of development in the rural Australian context, Holmes (2006) notes seven ‘modes of occupancy’ that capture a range of land uses and actor groups that increasingly seek to achieve (and sometimes impose) their own visions and goals within specific spatial settings. This conceptualization points to the widely competing demands on rural lands, not just for production, but for an array of ecosystem services, livelihood opportunities, amenity, experience, heritage preservation and many other purposes (Hart et al., 2015; Wilson, 2010). In this study, we use the terms multi-functional and multi-use interchangeably to describe a landscape conceptualization which includes more than one land-use designation. In the analysis itself we make a simple distinction between productivist and consumptivist land use activities and designations and expand our conceptualization of consumptivist land uses to capture 'resource protection' oriented designations. We do so as a matter of analytic convenience recognizing the frequency with which 'protected zone' designations are associated with passive consumption (see Fig. 1).

### Energy futures and spatial politics in rural areas

Representations of place and place identities are intersubjective and power-laden (Batel et al., 2015) and can operate as powerful levers of development control in energy transition (Batel 2020; Murphy, 2015). Shared visions of place are invoked and mobilized in the discourses that shape conversations about ‘preferred’ energy futures in particular places (Batel, 2020; Eaton et al., 2019; Fast and Mabee, 2015; Rudolph and Kirkegaard, 2019) and come to matter in framing (the articulation of) energy transition in-place (cf. Wolsink, 2020). More specifically, dominant visions of place become codified into territorial structures, most notably land-use plans, that shape processes of energy transition on the ground; e.g., in terms of determining which locations are deemed ‘acceptable’ or not for particular kinds of RE infrastructure (Calvert et al., 2019; Cowell, 2010).

Rural land-use plans are therefore significant sites of (potential) resistance and disassembly in the process of energy transition. Land-use plans institutionalize and codify place attachments and identities that act as levers of control on development (Cadieux et al., 2013; Crawford and French, 2008). Therefore, energy transition occurs alongside transformational change in the (dominant) discourses that come to define and shape places. This raises questions such as: How are rural land-use plans responding to the transition toward renewable energy? What is the relationship (if any) between how rural areas are represented/imagined, and how energy transitions are addressed, in rural land-use planning documents? Are discussions and discourses of energy transition entangled with, or kept separate from, narratives about rural change in official planning documents?

These questions are central to understanding how energy transitions and rural transformation are being co-constructed – and so far we have only partial answers. Naumann and Rudolph (2020: 100) suggest that ‘rural manifestations of energy transitions are accompanied by a reversal of the post-productive discourse, incorporating rural areas into new production and capital accumulation processes’. In a case study analysis, for instance, Woods (2003) finds a correlation between an individual’s support for a local wind farm and their tendency to promote a discourse of the ‘rural as a productive space’ – i.e. as a place to make a living; as a place for work and commodity production (see also Chappell et al., 2020). On the other hand, those who express opposition to the local wind farm are more likely to espouse the discourse of the ‘rural as a consumptive space’ – as a place of leisure and tranquillity (see also Phadke, 2011). Sæþórsdóttir and Ólafsdóttir (2020) study wind development in a rural tourist region and find that residents who live and make a living in these places are more supportive of wind energy than tourists who treat the area as a ‘playground’. Indeed, the politics of rurality, often interpreted through this ‘productivist-consumptivist’ lens, are ignited by, and folded into, debates about RE development, as for example when ‘…disputes over wind turbines aggravate social class divisions between those that see rural areas as landscape and those that see it as a place of production’ (Fast, 2015: 859).

Much of the work to date has interpreted politics of place in rural areas through a ‘productivist-consumptivist’ lens. This work has taken an individualist or socio-cognitive approach to studying place attachments and identities in rural areas; leaving out critical analyses of the territorial structures (land-use plans) that aim to control development in rural areas. If we extrapolate findings from individual to intersubjective framings, however, the work suggests that the orientation of landscape conceptualizations in land-use plans – as productive or consumptive (post-productive) – is linked to pathways of economic transformations and to sentiment toward RE more generally (see Figure 1). That is, we expect land-use plans that conceptualize landscape as a productive resource to also include more favourable references toward RE. On the other hand, we expect land-use plans that conceptualize landscape as amenity to include less favourable references to RE. Does this relationship hold within rural land-use planning documents in Ontario, Canada? And what about ideas of multi-functionality which seem to be glossed over when interpreting the spatial politics of rurality in terms of a productive-consumptive dichotomy?

## Case study and methods

In our attempt to understand the unfolding dynamics around RE in (mostly rural) Southern Ontario, we acknowledge that ‘place matters’. The Southern Ontario setting brings together some well recognized elements of the multi-functional rural landscape(s) noted above – and with them some of the antecedents for contestation around facility siting in the context of RE development noted in other regions. Though only partial and greatly simplified, we note the following broad situational factors as being relevant to the physical setting(s) and the socio-political ‘climate’ for RE development in the region. Southern Ontario is, at once:

- a region of rapid and intensifying urbanization and urban expansion, centering on and emanating outward from the so-called Greater Golden Horseshoe Region – a zone encompassing the Greater Toronto Area (GTA) extending westward across the north shore of Lake Ontario and around its western tip through the Niagara Region to the Canada/US border (Ontario Ministry of Municipal Affairs, 2017). Based on recent projections, population in the GGH is expected to reach 13.5 million by 2041, an increase of nearly 30% over the current population of just over 9 million (Ontario Ministry of Finance, 2021).
- a nationally and provincially important zone of agricultural production owing in no small part to the region containing the largest fraction of class 1–3 agricultural soils in the Country as rated by the Canada Land Inventory land classification system (Hoffman, 1971). Indeed, class 1 soils make up less than 1% of Canada's agricultural soils, and the greatest concentration of these (51%) is located in the Southwestern portion of the study area (Statistics Canada, 2001). Agriculture, both crop and livestock, with different scales and levels of intensity comprises the largest single land use category in the region and remains a sector of considerable economic importance with a strong voice in the socio-political realm (Ontario Ministry of Agriculture, Food and Rural Affairs, 2017).
- imbued with significant natural beauty and both ecological and hydrological significance deriving from its location in the Great Lakes Basin, surrounded by 3 of the Great lakes (Ontario, Erie and Huron) and with a moderate climate that supports ecological features and communities distinctive in Canada – especially across the northwestern portion of the Lake Erie shoreline (Ontario Ministry of Natural Resources, 2009). Such features serve as a focal point for both amenity-led development or ‘consumption’ activities on one hand and conservation/protection efforts on the other.
- and a region with a long established but now evolving settlement pattern where, beyond the long shadow of Toronto, many mid-sized urban centres are experiencing growth as a result of exurban migration from the Toronto market together with international immigration; while other smaller centres, including many traditional agricultural service communities, are seeking pathways to sustainability through diversification. In some instances, particularly for communities adjacent or proximate to one of the Great Lake these changes are driven by, and reflected in, the arrival of lifestyle seeking urbanites and the development of new forms of economic activity associated with amenity and the consumption economy Caldwell, 2018; Ontario Ministry of Finance, 2021).

So what for the Provincial energy sector, and the prospect of a successful (at least partial) transition to RE? The energy sector constitutes a spatially diffuse socio-technical system that includes not just the ‘hard’ components of energy production (natural resource assets and infrastructure development) but also the human-side elements that are reflected in societal values, public/consumer demands, public policy and related institutional arrangements, and the discourses that arise concerning the benefits and costs (often expressed in relation to place-specific outcomes) associated with different energy development alternatives (Li et al, 2015; Verbong and Geels, 2010). The attributes noted above are relevant to how future energy transitions might play out in Ontario in so far as they speak to the causes of increased energy demand, the environmental assets that might be implicated in new RE projects, and the mixed voices and values that will surround such development and seek entry into the process. In this paper we identify the land use planning process as a potential means for such participation. 

Rural Municipalities in Ontario are governed over the long-term by their ‘Official Plan’ (OP), a planning policy document that sets out the values and goals of the municipality and establishes policies and regulations to achieve them. Under the *Planning Act* of the Province of Ontario, every municipality is required to update their OP every five years and to operate on a 20- to 30-year planning horizon. Municipal OPs shape long-term landscape-level outcomes at the direction of and within the constraints set by the Provincial Planning Act. The planning intent expressed in an OP is applied through zoning amendments, minor variances, secondary plans and so on. Consequently, land-intensive/landscape-level RE transitions will be facilitated or hindered depending on the prevalence and strength of both RE transition themes and the characterization of the present and future landscape in OPs. In the context of RE planning, this may represent a novel element in the land-use planning process at the local level as, historically, energy planning and energy resource development is primarily Provincial jurisdiction. Rural communities and local planners across Ontario have had very little interaction with energy planning practices. Analysing these plans can help to determine the extent to which place values and attachments via landscape conceptualizations of production, consumption or multi-use are shaping energy and RE transitions planning.

**Figure 2.**
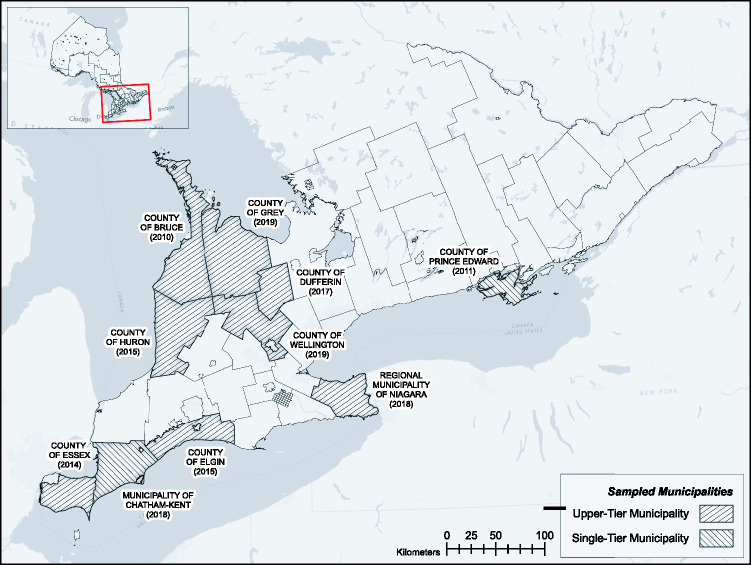
Sampled municipalities within Southern Ontario, along with the date at which their OP was adopted and/or updated. Upper-tier municipalities consist of two or more lower-tier municipalities and governance responsibilities are split between these two levels. Single-tier municipalities perform all governance responsibilities. Source: Reproduced with permission from Ontario Ministry of Municipal Affairs and Housing (2020a, 2020b).

The present study is based on a structured content analysis of OPs across 10 select municipalities in rural and peri-rural southwestern, central and eastern Ontario (see Figure 2). This represents 48% of all municipalities in southern Ontario classified as ‘rural’ according to Ahmed (2019), a classification scheme admittedly insensitive to the representational elements of rurality. Our sample is selected primarily from the southwest region, which hosts most of Ontario’s agricultural economy, and was purposively designed to include rural areas that are generally considered ‘commodity-focussed’ (heavy rotations of grain corn and soybeans) as well as rural areas that are considered to be more amenity focussed, in particular, Ontario’s emerging and rapidly expanding grape and wine regions. Digital copies of the most recently published OPs of the selected municipalities were collated and added to the qualitative content analysis software NVivo. This software effectively allows users to measure and analyse content in a document or file. Structured content analysis was performed in three steps: developing themes, coding the OPs and thematic analysis.

Our analysis began with a preliminary search of keywords which, collectively, define the initial themes/nodes of our codebook (Bernard, 2000; Neuendorf, 2017). Keywords were split across two categories or primary thematic nodes: energy transitions and place-making (see Table 1). Primary energy transition nodes identify explicit references to *Framing Terminology* (high-level strategic directions), *Energy Infrastructure* and *Energy Governance*. Primary place-making nodes identify explicit and implicit^1^ references that signal a rural discourse or a particular conceptualization of rural landscapes. Based on our above literature review, this category was partitioned between *Consumptivist Landscape Expressions* and *Productivist Landscape Expressions* (see also Frank and Hibbard, 2016; Mitchell, 2013).

**Table 1.** Primary thematic nodes that guided the content analysis.

Primary thematic node	Definition	References/secondary nodes
*Energy transition nodes*		
Framing terminology	Explicit references within an Official Plan to initiatives/intentions that seek to replace existing fossil fuel energy sources with renewable and non-renewable alternative energy sources.	Explicit references to: energy transition, energy transformation, low(-)carbon, climate change (mitigation)
Energy infrastructure	Elements of Official Plans that relate to the physical infrastructure associated with energy production. This includes projects and plans that significantly reduce the greenhouse gas emissions associated with specific energy infrastructure.	Explicit references to: renewable energy, alternative energy, solar, wind, hydro(electric), geothermal, nuclear, biogas
Energy governance	Elements of Official Plans that affect the non-physical elements of energy infrastructure, or the conditions under which energy infrastructure will be developed; especially components that affect the distribution of political power within energy governance.	Explicit references to: direct authority (how the OP will govern energy transition/infrastructure); a separate/secondary community or corporate energy plan (see Macdonald and Murphy, 2008); or relevant Provincial policies/legislation
*Place-making nodes*		
Consumptivist landscape expressions	Narratives in Official Plans that emphasize experiential activities drawing on the appeal of rural areas/countryside. Built features, landscape and sense of community are marketed.	Explicit and implicit references to: aesthetics and rural lifestyle (including rural idyll), cultural heritage, leisure (including parks and trails), tourism (including agri-tourism)
Productivist landscape expressions	Narratives in Official Plans that emphasize productive activities. Resources, commodity production and commercial/industrial activities are marketed.	Explicit and implicit references to: commodity agriculture; commercial and industrial activities; resource extraction

Once the primary themes and explicit references were developed, the OPs were read in full and coded, linking text to the preliminary codes. Coding was completed by two researchers to improve reliability.^2^ Code frequency is normalized to percent coverage of word count to enable comparisons across OPs since the length of OPs varied dramatically; the shortest document is approximately 60 pages, while the longest is approximately 400 pages.

Finally, a thematic analysis was conducted to determine the relationship between RE transition themes and the type of landscape conceptualization most prominent in each OP. The OPs were first positioned along the continuum noted in Figure 1, using a ratio of the relative prominence of productivist to consumptivist themes in their OP. A ratio higher than 1 indicates that these territorial structures conceptualize landscape as more productivist; and a ratio lower than 1 indicates that these territorial structures conceptualize landscape as more consumptivist. Once the OPs were organized using this ratio, we compared the prominence and character of energy transition codes to determine whether there is a link between how the rural landscape is conceptualized and how RE is treated.

## Results

### Energy transitions in rural Ontario OPs

**Figure 3.**
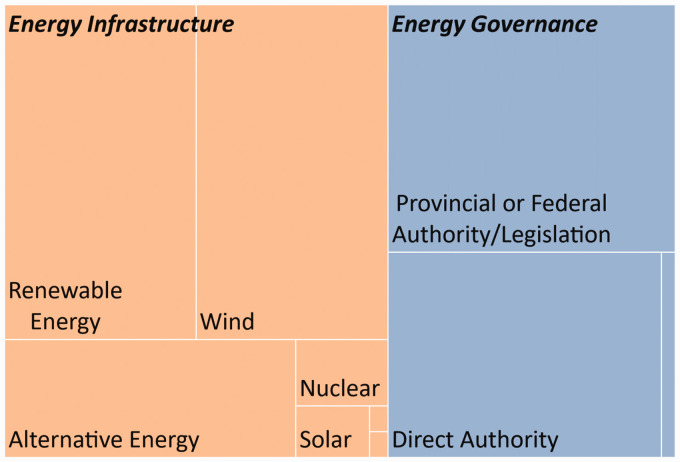
Average prevalence of renewable and alternative energy planning language by percent coverage across Official Plans of 10 rural municipalities in Southern Ontario. Note: This figure only illustrates relative prevalence and not how the planning language terms relate to one another. Refer to Table 1 for the hierarchical organization of themes and nodes.

Figure 3 provides a breakdown of the prevalence of key energy transition themes across all OPs. As a rule, the process of energy transition is not framed strategically in the OPs sampled for this study. That is, the sampled OPs did not contain any framing terminology for energy transitions (see Table 1 for search terms) in either the general planning objectives or specific planning policies – a noteworthy discovery in its own right. Instead, OPs refer to energy infrastructure more specifically. Even here, references are relatively scarce. On average, municipalities dedicate 0.35% of their OPs to alternative energy infrastructure, which includes both renewable and non-renewable alternatives to carbon-based energy infrastructure. In only one municipality – Niagara –we find a separate section of the OP dedicated to RE infrastructure, and it focuses exclusively on controls over wind turbine development. We note in passing that the Niagara region generally, and the portion of the region in close proximity to the Niagara River (and Niagara Falls) is amongst Canada’s most frequently visited and best known visitor destinations on the basis of both its natural features and a highly developed wine sector featuring both wine products and related ‘experience’.

More often than not, references to energy governance deflect attention to provincial policies/legislation. For example:

Regional Wind Energy policies … are to be interpreted and implemented in accordance with the provisions of [provincial energy legislation]. (Regional Municipality of Niagara, 2018: 8–10)

Alternative energy systems and renewable energy systems shall be permitted throughout the County in accordance with provincial legislation. (County of Huron Planning and Development Department, 2015: 14)

References to this type of ‘upscaling’ are observed more frequently than references to any form of local responsibility or local goals through land-use planning or other policy domains. Recently, municipalities across Ontario have engaged in a relatively new practice of ‘community energy planning’ which do not carry any authority or development control without being embedded into an OP. Only 1 of our 10 OPs makes any mention of a community energy plan.

In sum, planning for an energy transition remains limited and inconsistent across the rural municipalities sampled here. OPs focus on energy infrastructure and not on the overall emergence of any manner of energy transition and shifting energy landscapes. In the majority of cases, mentions of energy infrastructure are associated with references that deflect attention to provincial policies; local-level initiatives are very rare.

### Rural landscape conceptualizations

**Figure 4.**
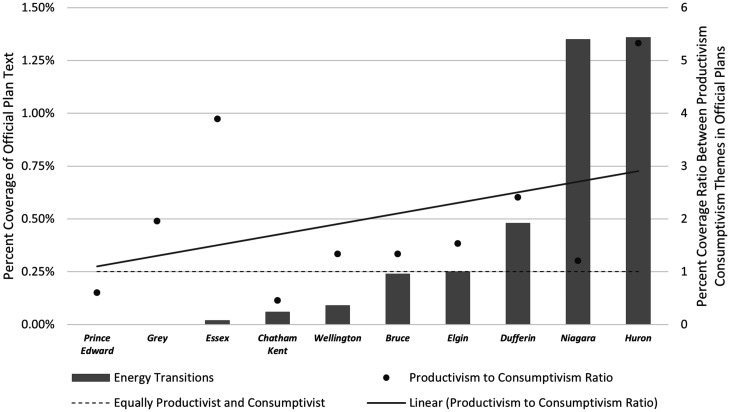
Prevalence of landscape productivism themes as measured by the percent coverage ratio between productivist- and amenity-landscape themes across the Official Plans of 10 rural municipalities in Southern Ontario (right axis). The total percent coverage of energy transition themes within those same plans (left axis). See Appendix A for data sources.

All OPs include language that conceptualize their landscapes as mixed-use – i.e. they all invoke narratives and activities associated with productivist and consumptivist landscape conceptualizations. That said, a ratio of productivist to consumptivist themes in OP demonstrates the overall tendency to conceptualize and plan for productivist landscapes more than consumptivist landscapes (see Figure 4). Productivism in planning is perhaps best exemplified by the County of Huron’s OP:

Agriculture in Huron is of national significance. Huron leads all counties and regions in Ontario in total value of production; and it also exceeds the production totals of several provinces. Huron has the advantage of an informed and progressive farm community, a supportive service sector, high capability soils, a diversified agricultural industry, a favourable climate, and limited nonfarm intrusion. (County of Huron Planning and Development Department, 2015: 6)

Just as the Niagara Region possesses and promotes a strong identity around landscape amenity and the wine sector, Huron County has long been seen as an agricultural heartland within the Ontario farm sector. Notably, land-use planning over time in the County of Huron has long exhibited concerted regard for the preservation of agricultural land and the County, and its approach in land-use planning, is seen as a Provincial and even National leader in this regard.

Generally, OPs included fewer policies for or references to consumptive land-use activities. Only two municipalities – Chatham-Kent and Prince Edward – have a greater percent coverage of consumptivist themes over productivist themes, which seem to revolve around attracting tourists and amenity migrants. Notably, the entirety of Prince Edward and the Lake Erie adjacent portion of Chatham-Kent are within a grape/wine region and one of the Province’s three recognized wine appellations. For example:

Prince Edward County will be a tranquil and beautiful place to live and visit. It will be unique from most parts of the Province because of its combination of natural beauty, heritage and rural charm. (Ainley and Associates Limited: Consulting Engineers and Planners and County of Prince Edward Planning and Development Committee and Planning Department, 2011: 10)

To preserve the scenic beauty and amenities of the area, extractive operations shall be screened from public view wherever possible. Screening may be provided by planting, fences and/or landscaped berms. (Municipality of Chatham-Kent, 2018: 3–41)

### Linking rural landscape conceptualizations and energy transitions

Due to the profound implications of RE development on landscape form and function, and on the basis of previous research into the factors underlying individual attitudes toward RE, we expected to see a link between rural landscape conceptualizations and RE transitions. Overall, this relationship is weak (see Figure 4). Rural municipalities that dedicate a greater proportion of their OP to productivist landscapes also dedicated a greater proportion to renewable and alternative energy, which indicates some relationship. Furthermore, the two municipalities with consumptivist-dominant narratives in their OP – Chatham-Kent and Prince Edward – are among the municipalities with the least amount of planning language related to energy transitions. In fact, Prince Edward, located on the eastern end of Lake Ontario with prevailing winds from the west, suggesting at least a site/situation suitability for wind power generation, included zero references to energy transition themes including RE infrastructure. Chatham-Kent’s OP dedicates only 0.06% to energy transition themes; and many of those references are weakly connected to RE generation and provide little incentive or direction for energy futures in the region. For example:

It is recognized that how energy is used and managed is directly linked to a healthy, reliable and sustainable energy future for Chatham-Kent. (Municipality of Chatham-Kent, 2018: 2–81)

However, the correlation between landscape conceptualization and energy transition is neither strong nor generalizable. Niagara, for instance, has the second-highest percent coverage of RE transition themes in their OP despite having nearly equal proportions of productivism and amenity landscape themes. Essex, meanwhile, has the second highest proportion of productivist to consumptivist narratives, and yet barely mentions energy transition.

It is also important to note that our findings are not always representative of ‘realities on the ground’. The OP guiding development in Prince Edward, for example, leans strongly toward consumptivist framings and is silent on RE development despite strong RE potential. This reflects ‘on the ground’ realities in the region: community opposition to wind energy development has been aggressive throughout the community and within the local government, culminating in highly coordinated legal battles against proposed wind farms. Chatham-Kent also leans strongly toward consumptivist framings and has relatively little to say about energy transition. In this case, however, ‘on the ground’ realities are very different. The area contains a significant amount of RE development which, although subject to local opposition (as are all projects, in one way or another), has not been subject to the highly coordinated attacks as seen in Prince Edward and has in fact received endorsement through other municipal channels such as regional economic development initiatives.

## Discussion

On the basis of previous research, we expected to see a relationship between discourses of rurality and of energy transitions across our study region. More specifically, we expected that OPs with the greatest emphasis on ‘productivist’ representations would also be more likely to incorporate energy transitions and energy infrastructure into these representations and planning documents (see Eaton et al., 2019). This expectation is based on the understanding that the orientation of landscape use in rural communities is linked to pathways of economic transformations: rural economies pursuing productive landscape transformation rely on the landscape itself as a productive resource to harvest or extract commodities, RE technologies being another in a long line of productive activities. On the other hand, rural economies pursuing amenity landscape transformations rely on the aesthetic of a rural landscape to produce tourism commodities, based in leisure or heritage landscapes; RE being an unwelcomed and unfit entrant. This expectation was not strongly supported in rural land-use plans across southern Ontario.

All OPs examined here show some efforts at balancing productivist and consumptivist narratives in their OPs (though, as ratios in Figure 4 show, some tilt the balance more than others). This demonstrates flexibility and an aim to be representative of the kinds of land-use practices and landscape values that constitute these places. Implicitly, this balancing infers a multi-functional land-use approach in these various municipalities. That said, the lack of framing terminology for energy transition is concerning. It suggests that the communities and officials who work to develop these plans have not identified energy transition as a driver of landscape transformation – at least not yet in the form of clearly articulated statements of intent or policy. It also suggests that local and community energy plans have not yet made substantive impact on official land-use planning practices, even as the goals and objectives expressed in energy plans are very clearly implicated in land-use planning.

One reason for this lack of integration could be legacy governance dynamics. In Canada, energy planning and energy resource development is primarily Provincial jurisdiction. As observed earlier, rural communities and local planners across Ontario have had very little interaction with energy planning practices. As the transition to localized and land-intensive energy systems unfolds, however, energy planning *becomes* land-use planning, and therefore, energy planning becomes a significant local-level planning domain. Local planners and communities are still figuring out their role in this regard.

The learning curve has been stunted by past Provincial legislation around RE development. The time period in which the OPs sampled in this study were developed or operational coincides with the *Provincial Green Energy and Green Economy Act* which revised the *Planning Act* to remove municipal powers on RE development and, instead, to centralize authority in a Provincial office as a way of providing a more consistent set of rules related to setback distances, zoning regulations and other matters that would have otherwise fallen to each individual municipality. The provincial objective was to treat RE development as an industrial policy – to stimulate rapid development and, in turn, investments into infrastructure and manufacturing capacity, by limiting the ability of local opposition to delay projects (see also Fast and Mabee, 2015). In effect, the areas deemed ‘acceptable’ for RE development were those that held provincial significance rather than local significance, similar to what has been observed in other regions (Cowell, 2010). In addition, ‘upscaling’ authority in this way incentivized municipalities to respond defensively – and de-incentivized proactive integration of energy into their land-use planning processes – processes that intentionally and explicitly solicit the participation of the public. A critical reflection of our methodology here might be to speculate how the outcomes of our study may have been different had we used older municipal land-use plans created before the GEGEA instead of using the current OPs which were developed or operational after the *Planning Act* revision.

As of 2018, the provincial context has changed. The GEGEA has been repealed and authority to manage RE development has been returned to municipalities, presumably through land-use plans, by-laws and zoning. As such, our analysis, though preliminary in nature, establishes a baseline which can be used to contrast changes to OPs as municipalities in Ontario begin to engage in local RE governance with greater authority. Other research has shown that local land-use plans can prioritize landscape protection and hamper development (Ohl and Eichhorn, 2010), but that remains to be seen in this case. Here, we note an opportunity for future research to investigate further and more thoroughly the links between rural landscape conceptualizations and energy transitions using discourse analysis. The analysis undertaken in this paper, together with the back story of the political context existing around RE development at the time most planning documents were prepared, tells us that we are, perhaps, positioned to catch the RE transition in Ontario at an important inflection point. With the return of at least partial energy planning authority to local municipalities, there exists the opportunity to observe the growth and integration of RE planning into the local land-use planning process in real time via a longitudinal approach. In this sense, Ontario may prove to be an instructive and revealing laboratory for observing the interplay between trajectories of development in the countryside and the inclusion (or not) of the RE portfolio as part of that shift.

## Conclusion

Rural land-use plans codify dominant and intersubjective representations of place and spatial identities and in turn help us to understand the kinds of place-meanings and identities that are being advocated and protected through territorial structures. Rural land-use plans conceptualize rural landscapes and rural places in non-neutral, partial ways: they highlight particular affordances or qualities – which we have broadly referred to as productivist, consumptivist and/or mixed-use – against which and through which energy transitions are interpreted and managed. Examining the links between land-use plans and energy transition, and the integration (or not) of energy planning with land-use planning, is therefore critical to our understanding of the contested and evolving geographies of energy transition more generally.

The research has opened up rural land-use plans as significant sites of (potential) resistance and disassembly in the process of energy transition. By treating ‘the rural’ as a socio-spatial process rather than a category with particular essential qualities, we are opened up to new questions about who selects the particular affordances/qualities of rural areas over others in the framing of energy transition; and we are forced to recognize the always contingent and power-laden nature of the place-meanings and identities that shape our individual and collective responses to energy transition (among many other kinds of structural change).

Finally, and somewhat speculatively, we note the potential for complementarity and collaboration, rather than contestation, in a RE-infused rural transition. One important question moving forward will be the extent to which, if at all, RE is at some point valorized and appropriated into rural discourses and framings, whether amenity-based, multifunctional or productive. It remains to be seen whether the potential ‘re-localization’ of RE planning and projects, with a greater measure of local control (in both reality and perception) may precipitate a different dynamic. Although work to date has focussed more on the determinants of RE acceptance by communities, a different and intriguing question is whether and how the RE sector and other dimensions of transitioning rural places and regions may find common ground through what might be termed, ‘frame sharing’. While empirical evidence and related research is scarce, recent developments in the wine regions of Italy (Zambon et al., 2018) and for some years now in Dartmoor National Park in Southwest England (Dartmoor National Park Authority, 2019) have shown how the adoption of a common and unifying goal such as ‘sustainability’, self-sufficiency, carbon neutrality or other robust high-level qualities has anchored planning processes and formed a foundation for both ‘interpreting the symbolic’ (see also Eaton et al., 2019) and ‘implementing the material’ elements of development. With the recent return to municipal-level RE planning in Ontario, certainly there is an opportunity for such ‘frame sharing’ and complementarity. A challenge then exists for the land-use planning process and planners themselves to lead future change in a way that both recognizes and integrates to mutual advantage, the many and varied demands on changing rural places.

## Notes

1 Explicit coding refers to the identification themes present in text using predefined keywords without subjective interpretation, e.g. ‘energy transition’, ‘farm diversification’. Implicit coding identifies implied themes in text contextually based on inference, e.g. ‘promote features that attract visitors’ implies the theme ‘tourism’ (see Bernard, 2000; Hsieh and Shannon, 2017).

2 The average measure of agreement, here expressed as a Kappa value, across all nodes is considered substantial at 0.71; the average percentage of coding agreement between the researchers is 99.4%.

## Appendix: References for sampled Official Plans

Ainley and Associates Limited: Consulting Engineers and Planners and County of Prince Edward Planning and Development Committee and Planning Department (2011) County of Prince Edward Official Plan. Belleville, Ontario. Available at: www.thecounty.ca/county-government/departments/planning/official-plan/#current

County of Bruce (2010) County of Bruce Official Plan. Available at: https://brucecounty.on.ca/services/planning-development/bruce-county-official-plan

County of Elgin (2015) Official Plan of the County of Elgin. Available at: www.elgincounty.ca/planning/

County of Grey (2019) Recolour Grey: County of Grey Official Plan. Available at: www.grey.ca/programs-initiatives/recolour-grey

County of Huron Planning and Development Department (2015) Huron County Official Plan. Available at: www.huroncounty.ca/plandev/county-official-plan/

Dufferin County (2017) Dufferin County Official Plan. Available at: www.dufferincounty.ca/planning-development/official-plan-and-provincial-land-use-planning-policies

Jones Consulting Group Ltd and County of Essex (2014) Official Plan. Available at: www.countyofessex.ca/en/doing-business/planning-and-development.aspx#

Municipality of Chatham-Kent (2018) Chatham-Kent Official Plan: Action towards sustainability. Available at: www.chatham-kent.ca/business/planning-services/official-plan

Regional Municipality of Niagara (2018) Imagine Niagara. Available at: www.niagararegion.ca/living/icp/policy-plan.aspx

The Corporation of the County of Wellington (2019) County of Wellington: Official Plan. Available at: www.wellington.ca/en/resident-services/pl-landusepolicies.aspx#Wellington-County-Official-Plan
